# GSK2606414 Sensitizes ABCG2-Overexpressing Multidrug-Resistant Colorectal Cancer Cells to Chemotherapeutic Drugs

**DOI:** 10.3390/biomedicines11113103

**Published:** 2023-11-20

**Authors:** Ze-Zhong Yu, Bu-Qing Xu, Ying-Ying Wang, Peng-Wei Zhang, Yu-Bin Shu, Zhi Shi

**Affiliations:** Department of Cell Biology & Institute of Biomedicine, National Engineering Research Center of Genetic Medicine, State Key Laboratory of Bioactive Molecules and Druggability Assessment, MOE Key Laboratory of Tumor Molecular Biology, Guangdong Provincial Key Laboratory of Bioengineering Medicine, College of Life Science and Technology, Jinan University, Guangzhou 510632, China; yuzezhong@stu2020.jnu.edu.cn (Z.-Z.Y.); xbq1002@stu2021.jnu.edu.cn (B.-Q.X.); wangyingying@stu2021.jnu.edu.cn (Y.-Y.W.); pengwei@stu2020.jnu.edu.cn (P.-W.Z.); sub7@jnu.edu.cn (Y.-B.S.)

**Keywords:** GSK2606414, ABCG2, multidrug resistance, colorectal cancer

## Abstract

Colorectal cancer is a common malignant tumor. A major factor in the high mortality rate of colorectal cancer is the emergence of multidrug resistance (MDR). Overexpression of the *ABCG2* gene in cancer cells directly leads to MDR. Finding new inhibitors of ABCG2 may be an effective way to overcome drug resistance. We found that the compound GSK2606414 enhanced the sensitivity of the ABCG2 substrate to the chemotherapeutic drugs mitoxantrone and doxorubicin in ABCG2-overexpressing multidrug-resistant colorectal cancer cells by increasing their intracellular accumulation without affecting the protein expression of ABCG2. Molecular docking experiments predicted that GSK2606414 could stably bind in the drug-binding pocket of ABCG2. In conclusion, GSK2606414 can sensitize ABCG2-overexpressed multidrug-resistant colorectal cancer cells to chemotherapy drugs and can be used as a potential inhibitor of ABCG2.

## 1. Introduction

Multidrug resistance (MDR) of cancer cells refers to the resistance of cancer cells to a variety of anticancer drugs, which have different structures and mechanisms of action and seriously limit the clinical chemotherapy effect of cancer patients [1,2]. MDR of cancer cells during chemotherapy is associated with a variety of mechanisms, including enhanced drug drain, growth factors, genetic factors (gene mutations, amplification, and epigenetic changes), enhanced DNA repair capacity, enhanced heterobiotic metabolism, and so on [3]. Each of these mechanisms leads to a reduction in the therapeutic effectiveness of drug delivery, making cancer treatment more difficult. Drug outflow is an important factor in the development of MDR in tumors. The ATP-binding cassette (ABC) transporter family, including ABCG2, ABCB1, etc., is a key drug efflux protein. Overexpression of ABCG2 protein (also known as breast cancer resistance protein (BCRP)) is a pivotal factor in generating MDR [4,5,6]. ABCG2, as one of the most common ABC transporters, is an “ABC half transporter” working as a homodimer in the cell membrane to obtain energy through ATP hydrolysis, thereby pumping substrates out of cells. The human *ABCG2* gene is located on 4q22~23 with a length of 66 kb, composed of 16 exons ranging from 60 bp to 332 bp and 15 introns. Human ABCG2 protein is a 72-kDa protein consisting of 665 amino acid residues and contains an N-terminal ATP binding domain (NBD) and a C-terminal transmembrane domain (TMD) that is composed of six helices, whose structure is the half-size and configuration of many other ABC transporter family proteins comprising two NBDs and two TMDs [4,5,6]. ABCG2 is highly expressed in many normal tissues, including the liver, small intestine, colon, rectum, placenta, central nervous system, vasculature, and so on. Lower expression of ABCG2 occurs in the adrenal and thyroid glands, lung, and cerebral cortex, etc. The normal tissue distribution of ABCG2 indicates that the main physiological function of ABCG2 may play an important role in the regulation of biliary secretion and intestinal absorption of toxic metabolites or xenobiotics, as well as a protective role in the maternal–fetal barrier and the blood–brain barrier. ABCG2 on the cell membrane regulates the distribution, absorption, and excretion of a variety of compounds, which can protect cells from death caused by high intracellular drug concentrations. ABCG2 can also interfere with drug delivery, reducing its bioavailability, intracellular concentration, and blood–brain barrier switching [7,8]. Increasing the concentration of drugs to counteract MDR in cancer is one way, but it can also lead to other toxic side effects. In addition, due to the important role of ABCG2 in normal physiological conditions, direct inhibition of its expression may lead to some side effects. The identified ABCG2 substrate chemotherapeutic drugs include antimetabolites (i.e., 5-fluorouracil, cladribine, methotrexate, and trimetrexate), topoisomerase inhibitors (i.e., mitoxantrone, irinotecan, topotecan, epirubicin, and doxorubicin), tyrosine kinase inhibitors (i.e., gefitinib, erlotinib, nilotinib, lapatinib, fostamatinib, vandetanib, ponatinib, tivozanib, alectinib, bosutinib, tandutinib, imatinib, dasatinib, sorafenib, regorafenib, and sunitinib), and so on [7,8]. In addition to chemotherapeutics, drugs including cimetidine, glyburide, nimodipine, tacrolimus, novobiocin, prazosin, sulfasalazine, and rosuvastatin are ABCG2 substrates. The substrates of ABCG2 are also composed of conjugated organic anions, including sulfated or glucuronide conjugates and organic conjugates of drugs, xenobiotics, endogenous substances, and so on. High expression of ABCG2 has been reported in colorectal cancer and might be associated with the tumor response to 5-fluorouracil or irinotecan in colorectal cancer patients [9,10,11]. Inhibition of ABCG2 transport activity is a common strategy for overcoming MDR in cancer, and the combination of ABCG2 inhibitors can therefore increase the sensitivity of cancer cells to substrate chemotherapy drugs. To overcome ABCG2-mediating MDR in cancer, we and other groups have identified some ABCG2 inhibitors, such as fumitremorgin C and its derivative ko143, elacridar, AG1478, sildenafil, AZ32, and KU55933, etc. [12,13,14,15,16,17,18]. However, it is still necessary to develop new ABCG2 inhibitors.

In the present study, we found that the protein kinase R (PKR)-like ER kinase (PERK) inhibitor GSK2606414 is also an effective ABCG2 inhibitor and sensitizes ABCG2-overexpressing multidrug-resistant colorectal cancer cells to ABCG2 substrate chemotherapeutic drugs. Molecular docking experiments showed that GSK2606414 maintained a stable conformation through hydrophobic interactions with hydrophobic amino acids around ABCG2 due to its special chemical structure bound to the drug-binding pocket of ABCG2. GSK2606414 interacts with ABCG2 through the π-π bond and intermolecular halide bond to further stabilize its binding conformation. GSK2606414 is well known as a potent PERK-specific inhibitor, but this study is the first to show that GSK2606414 has a specific chemical structure that competitively inhibits ABCG2.

## 2. Methods and Materials 

### 2.1. Cell Culture and Reagents 

GSK2606414 (Cat. No.: 1337531-36-8) was obtained from Boer Chemical, Inc. (Shanghai, China). KU55933 (Cat. No.: 587871-26-9) was obtained from TargetMol Chemicals, Inc. (Shanghai, China). Mitoxantrone (Cat. No.: 70476-82-3) was obtained from D&B Biotech, Inc. (Shanghai, China). Doxorubicin (Cat. No.: A603456-0025) was obtained from Sangon Biotech, Inc. (Shanghai, China). Cisplatin (Cat. No.: AA1A8019B) was obtained from Qilu Pharmaceutical Co. (Jinan, China). Rhodamine 123 (Cat. No.: 62669-70-9) was obtained from Sigma-Aldrich (Shanghai, China) Trading Co. (Shanghai, China). Anti-ABCG2 antibody (Cat. No.: RLT0053) was obtained from Ruiying Biotech Co. (Wuxi, China). Anti-β-actin antibody (Cat. No.: SC-47778) was obtained from Santa Cruz Biotech, Inc. (Santa Cruz, CA, USA). The compound 3-(4,5-dimethylthiazol-yl)-2,5-diphenyl-tetrazolium bromide (MTT) (Cat. No.: 298-93-1) was obtained from Yuanye Biotech Co. (Shanghai, China). Human ABCG2-overexpressing MDR colorectal cancer cells, S1-M1-80 vector, and ABCG2-knockout cells, S1-M1-80 sgABCG2, were generated as previously described [15]. Briefly, S1-M1-80 cells were selected with puromycin (Cat. No.: A1113803) from Thermo Fisher Scientific, Inc. (Waltham, MA, USA) at a concentration of 30 μg/mL after infection with the viral supernatant of LentiCRISPRv2 vector (Cat. No.: 98290) from Addgene Co. (Watertown, MA, USA), which contains a targeting sequence from exon 3 of the human *ABCG2* gene. A monoclonal S1-M1-80 cell line with the stable knockout of ABCG2 was generated by single-cell culture. The sequencing results of genomic DNA PCR production showed that a “C” base was deleted in the target position of S1-M1-80 sgABCG2 cells in comparison to S1-M1-80 vector cells. Both cells were cultured in Dulbecco’s modified Eagle’s medium (DMEM) (Cat. No.: C11995500BT) with 10% fetal bovine serum (Cat. No.: 10270-106) from Thermo Fisher Scientific, Inc, (Waltham, MA, USA), penicillin (100 U/mL) (Cat. No.: A600135), and streptomycin (100 ng/mL) (Cat. No.: A610494) from Sangon Biotech Inc. (Shanghai, China) at 37 °C, 5% CO_2_. Cell culture dishes and plates were from NEST Biotech Co. (Wuxi, China).

### 2.2. Cytotoxicity Assay

The cytotoxicity of drugs in S1-M1-80 vector and S1-M1-80 sgABCG2 cells was determined using an MTT assay. Cells were cultured at 7000 cells per well in 96-well plates and treated with the indicated concentration of drugs for 68 h. MTT, at a final concentration of 500 μg/mL, was added to each well. After incubating with MTT for another 4 h and discarding the cell culture medium, 50 μL of DMSO were added to dissolve the formazan crystals. The absorbance was measured at 570 nm using the microplate reader BioTek Synergy H1 from Agilent Technologies, Inc. (Santa Clara, CA, USA). The concentration of 50% inhibitive concentration (IC_50_) was calculated from survival curves with the Bliss method, as previously described [19,20].

### 2.3. Drug Accumulation Assay

S1-M1-80 vector and S1-M1-80 sgABCG2 cells were cultured as 50,000 cells per well in 12-well plates and treated with the indicated concentration of GSK2606414 or KU55933 for 1 h. Mitoxantrone, doxorubicin, or rhodamine 123 at a final concentration of 10 μM were added to each well. After incubating with mitoxantrone, doxorubicin, or rhodamine 123 for another 2 h, the fluorescent images of these compounds in the cells were taken under a confocal microscope LSM900 from Carl Zeiss, Inc. (Oberkohen, Germany). Following that, the cells were harvested, washed with phosphate-buffered saline (PBS) three times, and analyzed with a CytoFLEX flow cytometer from Beckman Coulter, Inc. (Brea, CA, USA) to measure the fluorescence intensity. The detected excitation/emission wavelengths of mitoxantrone, doxorubicin, and rhodamine 123 were 635/661 nm, 470/560 nm, and 503/527 nm, respectively.

### 2.4. Western Blot Assay

S1-M1-80 vector cells were cultured at 50,000 cells per well in 12-well plates and treated with GSK2606414 3 μM for 24, 48, and 72 h. Cells were lysed in lysis buffer (1% NP-40, 0.5% sodium deoxycholate, 0.1% SDS, 10 ng/mL PMSF, 0.03% aprotinin, 1 µM sodium orthovanadate) at 4 °C for 30 min. Lysates were centrifuged for 10 min at 14,000× *g*, and supernatants were stored at −80 °C as whole cell extracts. Proteins were separated on 10% SDS-PAGE gels and transferred to polyvinylidene difluoride membranes. Membranes were blocked with 5% BSA and incubated with the indicated primary antibodies. Corresponding horseradish peroxidase-conjugated secondary antibodies were used against each primary antibody. Signals were detected with the ChemiDoc XRS chemiluminescent gel imaging system from Analytik Jena AG (Jena, Thuringia, Germany).

### 2.5. Docking Analysis

The three-dimensional crystal structure of human ABCG2 protein, which has been reported to have an active binding site, was obtained from the Protein Data Bank (PDB ID: 6vxi), and the three-dimensional chemical structure of GSK2606414 was obtained from PubChem (the U.S. National Library of Medicine). The molecular docking was performed with AutoDock Vina, and the results were visualized by PyMOL. The most stable pose with a top-scoring of the ABCG2 and GSK2606414 complex was selected.

### 2.6. Statistical Analysis

All statistical analyses were performed using the SPSS 20.0 statistical software package. The experimental results were repeated at least 3 times, and the differences were determined by using the Student’s *t*-test on GraphPad prism8.3.0. Data was shown as mean ± SD. The statistical significance was denoted as “*” *p* < 0.05 and “**” *p* < 0.01.

## 3. Results 

### 3.1. GSK2606414 Sensitizes ABCG2-Overexpressing Colorectal Cancer Cells to ABCG2-Substrate Chemotherapeutic Drugs

**Figure 1:**
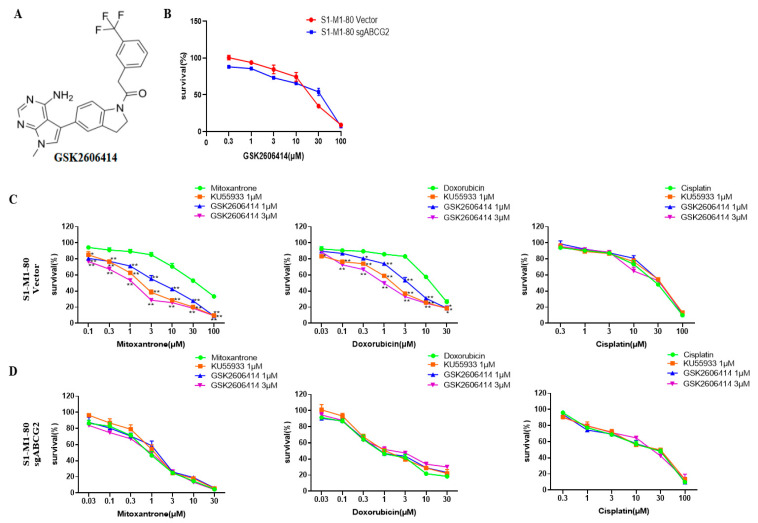
GSK2606414 sensitizes ABCG2-overexpressing colorectal cancer cells to ABCG2-substrate chemotherapeutic drugs. (**A**) The chemical structure of GSK2606414 is shown. Cells were treated with the indicated agents for 72 h and examined by MTT assay. The representative cell survival curves are shown (**B**–**D**).* *p* < 0.05, and ** *p* < 0.01 vs. the corresponding group.

To explore the effect of GSK2606414 (the chemical structure of which is shown in Figure 1A) on ABCG2-overexpressing colorectal cancer cells, cytotoxic assays of GSK2606414 were performed on both S1-M1-80 vector and S1-M1-80 sgABCG2 cells. As shown in Figure 1B, GSK2606414 showed dose-dependent cytotoxic effects on both cells. GSK2606414, at the low concentrations of 1 μM and 3 μM, was used to test its sensitizing effect. As shown in Table 1 and Figure 1C,D, GSK2606414 sensitized S1-M1-80 vector cells but not S1-M1-80 sgABCG2 cells to ABCG2-substrate chemotherapeutic drugs mitoxantrone and doxorubicin in a dose-dependent manner and was weaker than the known ABCG2 inhibitor KU55933 at the same concentration. Neither GSK2606414 nor KU55933 sensitized both S1-M1-80 vector and S1-M1-80 sgABCG2 cells to the non-ABCG2-substrate chemotherapeutic drug cisplatin. These data suggest that GSK2606414 can sensitize ABCG2-overexpressing colorectal cancer cells to ABCG2-substrate chemotherapeutic drugs. 

### 3.2. GSK2606414 Augments the Intracellular Levels of ABCG2 Substrates in ABCG2-Overexpressing Colorectal Cancer Cells

**Figure 2:**
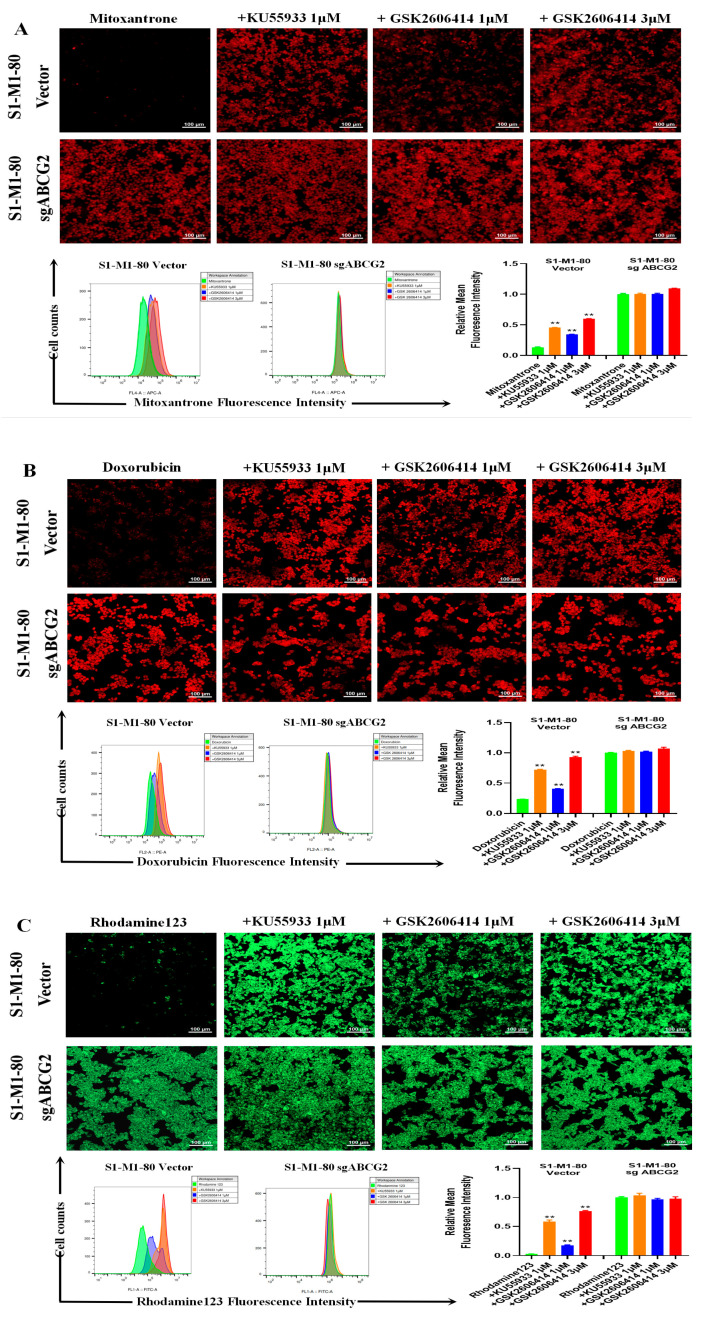
GSK2606414 augmented the intracellular levels of ABCG2 substrates in ABCG2-overexpressing colorectal cancer cells. Cells were incubated with 10 μM mitoxantrone, doxorubicin, or rhodamine 123 for another 2 h after pre-incubation with GSK2606414 or KU55933 for 1 h at 37 °C, photographed by confocal microscope and quantified by flow cytometer (**A**–**C**). ** *p* < 0.01 were compared with the corresponding group.

To investigate whether GSK2606414 can directly inhibit ABCG2 transporter activity, drug accumulation experiments were performed. As shown in Figure 2A–C, the intracellular levels of three ABCG2 substrates, mitoxantrone, doxorubicin, and rhodamine 123, in S1-M1-80 vector cells were lower than those in S1-M1-80 sgABCG2 cells. GSK2606414 dose-dependently enhanced the intracellular levels of mitoxantrone, doxorubicin, and rhodamine 123 in S1-M1-80 vector cells but not in S1-M1-80 sgABCG2 cells and was weaker than KU55933 at the same concentration. These results indicate that GSK2606414 can augment the intracellular levels of ABCG2 substrates in ABCG2-overexpressing colorectal cancer cells.

### 3.3. GSK2606414 Does Not Affect the Protein Expression of ABCG2 in Colorectal Cancer Cells and Binding Model of GSK2606414 with ABCG2

**Figure 3:**
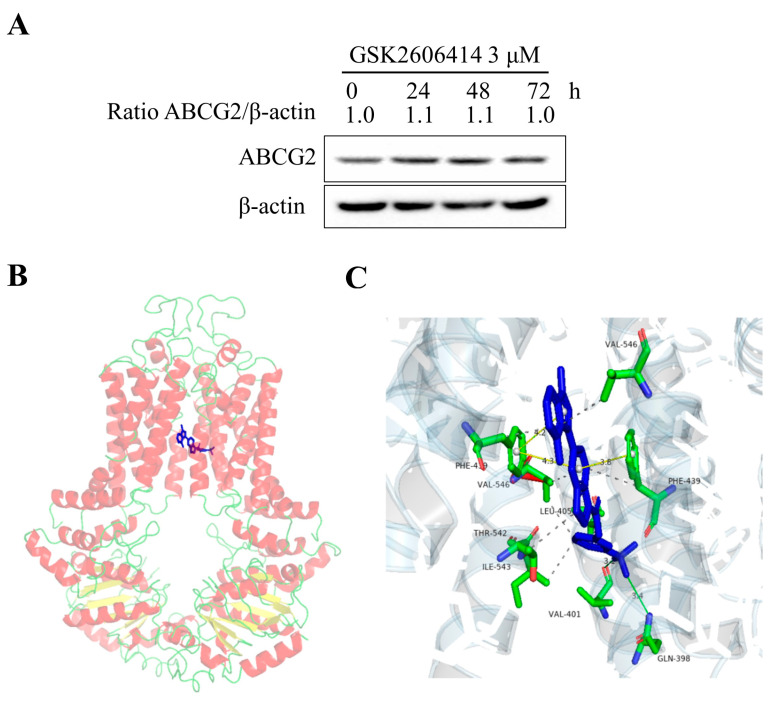
GSK2606414 does not affect the protein expression of ABCG2 in colorectal cancer cells and the binding model of GSK2606414 with ABCG2. (**A**) The ABCG2 expression level in S1-M1-80 vector cells treated with GSK2606414 3 μM for 24, 48, and 72 h was measured by Western blot assay. The optimal docked pose of GSK2606414 (blue sticks) within the drug-binding pocket of human ABCG2 is based on the three-dimensional crystal structure available from PDB (ID: 6vxi). ABCG2 conformation is presented as a ribbon diagram and colored according to secondary structure: red: helix, yellow: sheet, green: loop. GSK2606414 is shown as stick mode within a slit-like cavity of ABCG2, and the binding surface is exhibited as magenta (**B**). (**C**) Zoomed-in, the highlighted area shows that GSK2606414 interacts with the residues Gln398, Val-401, Leu-405, Ph-439, Val-546, Thr-542, and Ile-543 of ABCG2.

To detect whether GSK2606414 affects the expression of ABCG2 protein, S1-M1-80 vector cells were treated with GSK2606414 3 μM for 24, 48, and 72 h. As shown in Figure 3A, GSK2606414 did not affect the protein expression of ABCG2 in S1-M1-80 vector cells.

The revealed three-dimensional crystal structure of ABCG2 fosters the understanding of substrate or inhibitor recognition [21,22,23]. A molecular docking analysis was used to investigate the binding of GSK2606414 with ABCG2. The most stable pose with a top-scoring of the ABCG2 and GSK2606414 complex was selected. As shown in Figure 3A,B, GSK2606414 is located in the drug-binding pocket of ABCG2, and its conformation is stabilized by hydrophobic interaction with the surrounding hydrophobic amino acids such as Val-401, Leu-405, Val-546, Thr-542, and Ile-543. GSK2606414 is sandwiched between the benzene rings of Ph-439 on two monomers of ABCG2. The benzene ring of GSK2606414 interacted with Ph-439 by π-π bond, and GSK2606414 formed the intermolecular halide bond with Val-401 and Gln398 to further stabilize its binding conformation. In brief, GSK2606414 directly binds to ABCG2 with a special chemical structure connection to inhibit the pump activity of ABCG2.

## 4. Discussion 

GSK2606414, a highly potent ATP-competitive PERK inhibitor through targeting the inactive DFG (Asp-Phe-Gly) conformation in the ATP binding region of PERK, was developed and could inhibit the subcutaneous tumor xenograft growth of human pancreatic ductal adenocarcinoma (PDAC) cells BxPC3 [24], the organoid-based tumor xenografts growth of PDAC with high BZW1 level [25], and the phenotypic transition of cancer-associated fibroblasts in PDAC [26]. Inhibition of PERK by GSK2606414 was able to enhance simvastatin–temozolomide-induced cell death in human glioblastoma cells U87 [27], folate deficiency-induced cell apoptosis in human hepatocellular carcinoma cells HepG2 [28], and metformin-induced cell apoptosis in the human epithelial ovarian cancer cells PA-1 and OVCAR-3, but not in peripheral blood mononuclear cells and normal ovarian surface epithelial cells [29]. On the contrary, inhibition of PERK by GSK2606414 had protective effects on the photodynamic therapy of chlorin A-induced cell apoptosis in the human cholangiocarcinoma cells HuCCt1 and EGI-1 [30], heme oxygenase-1 inducer cobalt protoporphyrin, carbon monoxide donor CORM, microtubule disruptors taxol and nocodazole-induced cell apoptosis in human colon cancer cells COLO205 and HCT-15 [31,32], curcumin derivative WZ35-induced cell apoptosis in mouse colorectal cancer cells CT26 [33], and evodiamine-induced cell apoptosis in human renal cell carcinoma cells A498 [34], epithelial ovarian cancer cells A2780 and its cisplatin-resistant cells A2780CP [35]. During the development of intervertebral disc degeneration, the expression of inflammatory cytokines unfolded protein response (UPR) mediated by the PERK pathway was enhanced. The PERK inhibitor GSK2606414 inhibits the expression of endoplasmic reticulum stress-induced inflammatory cytokines such as tumor necrosis factor-α and IL-6 [36]. In addition, inhibition of PERK by GSK2606414 showed synergistic anticancer activity with digoxin against human leukemia cells K562 and THP-1 [37], and with the proteasome inhibitor bortezomib against human multiple myeloma cells H929 and L363 [38]. Moreover, inhibition of PERK by GSK2606414 caused an obvious reduction of the colony-forming ability in human lung adenocarcinoma cells with KRAS G12C (H358, H23) compared to WT KRAS (H1299, H1703) [39] and more cytotoxic effects on human breast cancer drug-resistant cells MCF-7-Epi^R^ and MCF-7-Tax^R^ compared to the parental MCF-7 [40]. Interestingly, inhibition of PERK by GSK2606414 promoted reovirus infection in human head and neck squamous cell carcinoma cells HN5 (tongue) and FaDu (hypopharynx) through an ATF4-dependent mechanism [41]. 

In addition to its inhibitory effect on a variety of tumors, GSK2606414 has a uniform improvement effect in neurodegenerative diseases such as Alzheimer’s disease, Parkinson’s disease, amyotrophic lateral sclerosis, and so on. These neurodegenerative diseases are associated with the accumulation and aggregation of misfolded disease-specific proteins in the brain. GSK2606414 could penetrate the blood–brain barrier, reverse cognitive deficits, and produce neuroprotective effects in prion-infected mice [42]. In addition, inhibition of PERK phosphorylation by GSK2606414 prevented translational inhibition in prion-infected mice and reduced the increase in ATF4 and CHOP caused by prion-mediated UPR activation [42]. GSK2606414 was able to inhibit PERK in the insular cortex, thereby reducing p-eIF2α levels and enhancing new taste learning and conditioned taste aversion [43]. PERK functions as a physiological constraint on memory consolidation in the cerebral cortex, and blocking PERK with GSK2606414 could enhance cognition. In Marinesco–Sjogren syndrome, GSK2606414 delayed the degeneration of purkinje cells and the onset of dyskinesia, prolonged the asymptomatic phase of the disease, and alleviated sports injury and skeletal muscle pathology during the symptomatic phase [44]. In Parkinson’s disease, chronic PERK signaling leads to apoptosis induction and neuronal dysfunction by inhibiting the translation of synaptic proteins. GSK2606414 pharmacologically blocked PERK to effectively inhibit ER stress stimulation while protecting substantia nigra dopaminergic neurons from Parkinson’s disease-induced neurotoxins, increasing dopamine levels and synaptic protein expression, and improving motor performance [45]. In a mouse model of frontotemporal dementia, neuron loss is caused by a brief shutdown of the PERK/eIF2α branch-mediated translation of UPR, of which mutant tau protein is one of the misfolded proteins. Treatment with the PERK inhibitor GSK2606414 restored the rate of protein synthesis in mice expressing mutated tau protein, reduced neuronal loss and brain atrophy, and eliminated the appearance of clinical symptoms [46]. GSK2606414 also improved neurotoxicity by inhibiting the PERK/p-eIF2α/ATF4/CHOP signaling axis and rescuing oxidative stress-induced mitochondrial dysfunction in N2A nerve cells exposed to high glucose [47]. Moreover, ER stress triggers up-regulation of apoptotic markers such as Bax, caspase-3, and CHOP, and GSK2606414 could significantly reduce the expression levels of these apoptotic markers to antagonize the protein changes induced by high glucose [47]. In addition, GSK2606414 inhibited PERK and negatively regulated osteoclast formation and bone resorption. GSK2606414 down-regulated the mRNA levels and protein expression of osteoclast differentiation marker genes and inhibited RANKL-induced mitogen-activated protein kinase (MAPK) and nuclear factor κB (NF-κB) pathway activation [48]. GSK2606414 was able to enhance glucose-stimulated insulin secretion, islet insulin content, and calcium transport in mice and human islets, suggesting that GSK2606414 is also a potential drug in the treatment of insulin deficiency. Additionally, GSK2606414 induced the expression of the ER chaperone BiP and the release of ER calcium [49].

In the current study, cytotoxicity experiments and drug accumulation assays were carried out in colorectal cancer cells with high ABCG2 expression and ABCG2 knockout, which directly demonstrated that GSK2606414 competitively inhibited the transport activity of ABCG2 in a dose-dependent manner and reversed cancer MDR in colorectal cancer cells with high ABCG2 expression. Our results of Western blot showed that the protein expression of ABCG2 was not affected by GSK2606414 treatment up to 72 h in colorectal cancer cells with high ABCG2 expression. These data suggest that GSK2606414 may be a new inhibitor of ABCG2 to enhance the sensitivity of the ABCG2 substrate chemotherapeutic drugs mitoxantrone and doxorubicin in ABCG2-overexpressing multidrug-resistant colorectal cancer cells by increasing their intracellular accumulation without affecting the protein expression of ABCG2. Molecular docking experiments also proved that GSK2606414 could stably bind to the ABCG2 transport pocket due to its chemical structure, and competitive binding of ABCG2 resulted in its inability to efficiently export substrate chemotherapy drugs, thus producing antagonistic effects on cancer MDR. GSK2606414 can competitively inhibit the drug activity of ABCG2 transport substrates due to its special chemical structure, most likely not because of its role as a PERK inhibitor. Similarly, several reports have shown that GSK2606414 can act in a PERK-independent manner. GSK2606414 was demonstrated to be a potent inhibitor of RIPK1 kinase [50,51]. GSK2606414 was also able to inhibit KIT tyrosine kinase activity and enhance endocytosis and lysosomal degradation [52]. However, the effects of GSK2606414 on the ATPase activity of ABCG2 and the sensitivity of ABCG2 substrate chemotherapeutic drugs in the treatment of colorectal cancer in vivo still need to be investigated in the future. 

## 5. Conclusions

In summary, GSK2606414 is a potential ABCG2 inhibitor and sensitizes ABCG2-overexpressing multidrug-resistant colorectal cancer cells to ABCG2-substate chemotherapeutic drugs. Our data provides a potential combination therapy strategy for overcoming MDR in colorectal cancer.

## Figures and Tables

**Table 1:** Summary of the IC_50_ values. The fold reversal value was calculated by dividing the IC_50_ of each drug in S1-M1-80 vector or S1-M1-80 sgABCG2 cells in the absence of inhibitors by that in the presence of inhibitors. ** *p* < 0.01 vs. the corresponding group.

	IC_50_ (μM) ± SD (Fold Reversal)	
Compounds	S1-M1-80 Vector	S1-M1-80 sgABCG2
Mitoxantrone	31.407 ± 2.119 (1.00)	0.911 ± 0.087 (1.00)
+KU55933 1 μM	2.012 ± 0.067 (15.61) **	1.106 ± 0.113 (1.21)
+GSK2606414 1 μM	5.080 ± 1.168 (6.18) **	0.904 ± 0.188 (0.99)
+GSK2606414 3 μM	1.289 ± 0.209 (24.36) **	0.904 ± 0.183 (0.99)
Doxorubicin	11.920 ± 0.605 (1.00)	0.776 ± 0.128 (1.00)
+KU55933 1 μM	1.562 ± 0.069 (7.63) **	0.919 ± 0.084 (1.18)
+GSK2606414 1 μM	3.403 ± 0.362 (3.50) **	0.836 ± 0.084 (1.08)
+GSK2606414 3 μM	1.179 ± 0.122 (10.11) **	0.975 ± 0.049 (1.26)
Cisplatin	28.933 ± 0.472 (1.00)	27.830 ± 1.682 (1.00)
+KU55933 1 μM	28.813 ± 1.259 (1.00)	28.000 ± 0.616 (1.01)
+GSK2606414 1 μM	28.053 ± 1.766 (1.03)	26.707 ± 1.499 (0.96)
+GSK2606414 3 μM	27.483 ± 2.283 (1.05)	27.047 ± 2.213 (0.97)

## Data Availability

Data is available upon reasonable request to the corresponding author.
